# Predictive value of *ACE* I/D genetic polymorphism for 12-month all-cause mortality in patients with acute myocardial infarction

**DOI:** 10.1097/MD.0000000000034976

**Published:** 2023-09-01

**Authors:** Duy Cong Tran, Minh Duc Do, Linh Hoang Gia Le, Truc Thanh Thai, Sy Van Hoang, Binh Quang Truong

**Affiliations:** a Department of Internal Medicine, University of Medicine and Pharmacy at Ho Chi Minh City, Ho Chi Minh City, Vietnam; b Department of Cardiology, Cho Ray Hospital, Ho Chi Minh City, Vietnam; c Cardiovascular Center, University Medical Center Ho Chi Minh City, Ho Chi Minh City, Vietnam; d Center for Molecular Biomedicine, University of Medicine and Pharmacy at Ho Chi Minh City, Ho Chi Minh City, Vietnam; e Faculty of Public Health, University of Medicine and Pharmacy at Ho Chi Minh City, Ho Chi Minh City, Vietnam.

**Keywords:** *ACE* I/D, acute myocardial infarction, all-cause mortality, genetic polymorphism

## Abstract

The prognostic role of the angiotensin-converting enzyme (*ACE*) insertion/deletion (I/D) genetic polymorphism in patients with acute myocardial infarction (AMI) is controversial and inconsistent across various study populations. This study evaluated the predictive validity of the *ACE* I/D variant based on 12-month all-cause mortality in Vietnamese patients after AMI. This was an observational, prospective study conducted among AMI patients at Cho Ray Hospital between January 2020 and September 2021. All participants were identified for *ACE* I/D polymorphism using the polymerase chain reaction method, with follow-up on survival status at 12 months from the date of admission. The proportions of II, ID, and DD genotypes of the *ACE* I/D variant were 49.5%, 35.9%, and 14.6%, respectively. All-cause mortality after 12 months occurred in 58 cases (10.6%). The *ACE* I/D polymorphism did not affect all-cause mortality in the dominant (*P* = .196), recessive (*P* = .827), homozygous (*P* = .515), and heterozygous (*P* = .184) models. A subgroup analysis by usage status of angiotensin-converting enzyme inhibitor/angiotensin II receptor blocker (ACEI/ARB) showed that in the non-ACEI/ARB group, patients with the DD genotype had a lower cumulative survival probability than patients with the II/ID genotypes (hazard ratio [HR] = 3.97, 95% confidence interval [CI]: 1.21–13.04; *P* = .023). Among patients with Global Registry of Acute Coronary Events (GRACE) scores below the median (153.5 points), those with DD genotype had a higher risk of mortality than those with the II/ID genotypes (HR = 3.35, 95% CI: 1.01–11.11; *P* = .049). The *ACE* I/D genetic polymorphism was found not to be associated with 12-month all-cause mortality in Vietnamese patients with AMI. However, it was associated with mortality in patients who did not use ACEI/ARB and also whose GRACE scores were below 153.5 points.

## 1. Introduction

Although diagnostic approaches, therapies, and prevention strategies have achieved much progress, coronary artery disease (CAD) remains the leading cause of mortality globally.^[1]^ Among the clinical types of CAD, acute myocardial infarction (AMI) causes most deaths, incurs considerable medical costs, and results in a significant socio-economic burden.^[2]^ The global burden of cardiovascular disease and AMI has shifted to low- and middle-income countries, which account for more than 3-quarters of global cardiovascular deaths.^[3]^ In addition, there is evidence that long-term mortality rates have not improved to the same extent as short-term mortality rates after AMI in recent decades.^[2]^

The prognosis of mortality in patients with AMI is influenced by many factors, such as demographic characteristics, comorbidities, clinical features, laboratory parameters, and treatment measures.^[4,5]^ Environmental components and genetic factors can also affect the mortality of AMI patients. Several genetic markers associated with AMI have been identified, including genes encoding components of the renin-angiotensin-aldosterone (RAA) system.^[6]^ The angiotensin-converting enzyme (*ACE*) gene is one of the genes in the RAA system that has been studied extensively in patients with AMI. In the *ACE* gene, the insertion/deletion (I/D) genetic polymorphism has been most commonly studied and has been shown to be associated with the risk and prognosis of AMI.^[7–11]^ *ACE* I/D is not a true variant, but an insertion or deletion of a 287 bp deoxyribonucleic acid (DNA) fragment located at intron 16 of chromosome 17.^[6]^ The DD genotype and D allele of the *ACE* I/D polymorphism are significantly associated with increased plasma ACE concentrations.^[12–14]^ An increase in ACE concentrations leads to a rise in angiotensin II levels with many physiological effects, such as vasoconstriction, aldosterone synthesis, salt and water retention, pro-inflammatory effects, and growth and remodeling of the myocardium and blood vessels.^[15]^

Since its discovery in 1990 using the polymerase chain reaction-restriction fragment length polymorphism technique, the *ACE* I/D genetic polymorphism has been revealed in several studies to be related to mortality in patients with AMI.^[7–11]^ In contrast, other studies have not found an association between *ACE* I/D and death in AMI.^[16–19]^ These conflicting results might be due to genetic differences between different countries, geographic regions, and races. Data on the association of *ACE* I/D with the prognosis of AMI is limited in Asian countries. Therefore, further studies of the *ACE* I/D variant may provide useful information for therapy individualization and optimization in AMI patients in the era of precision medicine. Thus, we conducted this study to investigate the prognostic role of the *ACE* I/D genetic polymorphism in the Vietnamese AMI population, thereby contributing to prevention and management strategies for AMI.

## 2. Methods

### 2.1. Study design and subjects

This prospective, observational study was carried out at Cho Ray Hospital, Ho Chi Minh City, Vietnam between January 1, 2020 and September 30, 2021. The Ethics Committee in Biomedical Research of the University of Medicine and Pharmacy at Ho Chi Minh City approved this research. The initial sample size was calculated to estimate the difference in the survival rates among different genotypes of *ACE* I/D polymorphism. A minimum of 33 deaths or 413 AMI patients were needed to have a statistical power of at least 80% with a 95% confidence interval to detect a hazard ratio (HR) of 2 or more.

Inclusion criteria were AMI patients ≥18 years of age who agreed to take part in the study. AMI was diagnosed according to the fourth universal definition of myocardial infarction.^[20]^ We excluded patients with a history of AMI, percutaneous coronary intervention, or coronary bypass surgery; with no contrast coronary angiography; with normal epicardial coronary artery branches on coronary angiography; or with whom contact was lost during follow-up.

The history of symptoms and signs was obtained for hospitalized patients, and physical examination and laboratory tests were performed by experienced cardiologists to confirm the diagnosis of AMI. Patients who fulfilled the sampling criteria were enrolled. Written informed consent was obtained from all participants. The data collected included demographic characteristics, coronary risk factors, clinical type of AMI, Killip class, troponin I concentration, estimated glomerular filtration rate, fasting lipid profile, and left ventricular ejection fraction (LVEF). All participants underwent coronary angiography and the location of stenosed coronary artery branches, the degree of coronary stenosis, and the number of diseased vessels were recorded. The Gensini score was used to assess the severity of CAD.^[21]^

Participant prognosis was evaluated using the Global Registry of Acute Coronary Events (GRACE) score on admission^[22]^ and therapeutic drugs at discharge were recorded. Patients were followed up for all-cause mortality in the hospital and every 3 months thereafter until 12 months from the date of admission. Survival or mortality was noted through regular rechecks, hospitalizations at Cho Ray Hospital, or telephone calls.

### 2.2. Genotyping

Each participant provided 2 mL of venous blood collected in EDTA-anticoagulant tubes. Genomic DNA was extracted from the peripheral leukocytes using the GeneJet TM Whole Blood Genomic DNA Purification Mini Kit (Thermo Fisher Scientific, Waltham, MA) according to the manufacturer instructions. The *ACE* I/D polymorphism was identified by polymerase chain reaction with Takara Taq polymerase (TakaraBio, San Jose, CA) in a SimpliAmp thermal cycler (Thermo Scientific) using the previously developed protocol.^[23]^ Fifty random DNA samples were directly sequenced by appropriate primers and the sequencing results were compared with corresponding polymerase chain reaction results to assure the accuracy of *ACE* I/D identification. The detailed protocol for direct sequencing were described elsewhere.^[24,25]^

### 2.3. Statistical analysis

SPSS 22.0 software for Windows (IBM, Armonk, NY) was used to process the study data. The normal distribution of continuous variables was determined based on the Kolmogorov-Smirnov test. Continuous variables were presented as mean ± standard deviation if normally distributed or median (interquartile range) if not normally distributed. Categorical variables were expressed as frequencies (percentages). Hardy-Weinberg equilibrium was evaluated by the Chi-squared test. The baseline characteristics of II, ID, and DD genotype groups were compared using the Chi-squared or Fisher exact tests for categorical variables and the ANOVA or the Kruskal–Wallis rank tests for continuous variables depending on data distribution. The differences between the factors in the deceased group and the survivor group were assessed using the Chi-squared or Fisher exact tests for categorical variables and the Student *t* test or Mann–Whitney *U* test for continuous variables depending on the variables’ standard distribution. The association between *ACE* I/D genetic polymorphism and 12-month all-cause mortality was analyzed using the Cox regression model in the dominant (II vs ID/DD), recessive (II/ID vs DD), homozygous (II vs DD), and heterozygous (II vs ID) inheritance models. The effect of the *ACE* I/D variant on mortality in subgroups with or without angiotensin-converting enzyme inhibitor/angiotensin II receptor blocker (ACEI/ARB) use and a GRACE score of less than or greater than the median was evaluated using the Cox regression model, and survival curves were estimated using the Kaplan–Meier method and compared using a log-rank test. The results of tests were considered statistically significant when the *P* value <.05.

## 3. Results

### 3.1. Baseline characteristics

There were 548 patients diagnosed with AMI recruited in the study. The frequencies and proportions of *ACE* I/D genotypes are presented in Table 1. *ACE* I/D genotypes in our study population were not at Hardy-Weinberg equilibrium (*P* < .001). The DD genotype has the lowest frequency (14.6%).

**Table 1:** Distribution of *ACE* I/D genotypes.

*ACE* I/D genotypes	Frequency	Percentage (%)
II	271	49.5
ID	197	35.9
DD	80	14.6
Total	548	100.0

ACE = angiotensin-converting enzyme, I/D = insertion/deletion.

Baseline characteristics of the overall population and subgroups by *ACE* I/D genotypes are presented in Table 2. The mean age of the study population was 63.9 ± 11.6 (range 30 to 90 years). Male patients were predominant at 71.5% of participants. The most frequent coronary risk factors were dyslipidemia (89.1%) and hypertension (81.8%). ST-segment elevation myocardial infarction (STEMI) was more common than non-STEMI (64.6% vs 35.4%). The majority of participants were Killip class I (76.6%), and the median LVEF was mildly reduced (47.0%). Regarding the characteristics of coronary artery lesions, triple vessel disease accounted for the highest proportion (40.9%). The median Gensini score was 37.0. Participants received standard therapy, including antiplatelet agents, statins, ACEI/ARB, and beta-blockers. The patients’ median GRACE score was high at 153.5 points. The subgroups with II, ID, and DD genotypes did not significantly differ in terms of clinical features, laboratory parameters, coronary artery lesions, and prognosis. Treatment characteristics were not statistically different between the II, ID, and DD genotype subgroups, except for usage of ACEI/ARB (93.0% vs 89.3% vs 82.5%, respectively; *P* = .019).

**Table 2:** Baseline characteristics of the study population according to *ACE* I/D genotypes.

Variables	Total (n = 548)	II (n = 271)	ID (n = 197)	DD (n = 80)	*P*
Clinical and laboratory parameters					
Age (yr), mean ± SD	63.9 ± 11.6	64.2 ± 12.1	63.5 ± 10.8	63.6 ± 12.2	.752
Male, n (%)	392 (71.5)	199 (73.4)	136 (69.0)	57 (71.2)	.581
Hypertension, n (%)	448 (81.8)	216 (79.7)	166 (84.3)	66 (82.5)	.444
Diabetes mellitus, n (%)	133 (24.3)	63 (23.2)	49 (24.9)	21 (26.2)	.834
Dyslipidemia, n (%)	489 (89.1)	239 (88.2)	175 (88.8)	75 (93.8)	.361
Obesity, n (%)	112 (20.4)	55 (20.3)	42 (21.3)	15 (18.8)	.888
Smoking, n (%)	233 (42.5)	121 (44.6)	83 (42.1)	29 (36.2)	.406
Family history of premature CAD, n (%)	37 (6.8)	20 (7.4)	14 (7.1)	3 (3.8)	.508
STEMI, n (%)	354 (64.6)	171 (63.1)	131 (66.5)	49 (61.2)	.640
Killip Class ≥ II, n (%)	128 (23.4)	63 (23.2)	49 (24.9)	17 (21.2)	.802
Troponin I on admission (pg/mL), median (IQR)	15.3 (3.1–50.1)	14.6 (3.7–50.0)	18.1 (2.0–50.0)	18.2 (1.5–50.0)	.746
eGFR (mL/min/1.73 m^2^), median (IQR)	83.1 (64.0–94.5)	83.2 (66.8–93.9)	84.3 (66.8–95.6)	76.2 (59.1–95.2)	.294
LVEF (%), median (IQR)	47.0 (39.0–53.0)	47.0 (40.0–53.0)	46.0 (38.0–53.0)	46.5 (35.0–52.8)	.629
Coronary lesions					
Single vessel disease, n (%)	133 (24.2)	66 (24.4)	47 (23.9)	20 (25.0)	.998
Double vessel disease, n (%)	191 (34.9)	93 (34.3)	70 (35.5)	28 (35.0)	
Triple vessel disease, n (%)	224 (40.9)	112 (41.3)	80 (40.6)	32 (40.0)	
Left main disease, n (%)	54 (9.9)	26 (9.6)	20 (10.2)	8 (10.0)	.979
Gensini score, median (IQR)	34.0 (17.3–58.0)	34.0 (18.0–60.0)	34.0 (16.0–52.0)	40.0 (19.5–73.5)	.048
Medications at discharge					
Aspirin, n (%)	547 (99.8)	270 (99.6)	197 (100.0)	80 (100.0)	.494
P2Y12 inhibitor, n (%)	548 (100.0)	271 (100.0)	197 (100.0)	80 (100.0)	-
Statins, n (%)	540 (98.5)	268 (98.9)	193 (98.0)	79 (98.8)	.712
ACEI/ARB, n (%)	494 (90.1)	252 (93.0)	176 (89.3)	66 (82.5)	**.019**
Beta-blocker, n (%)	409 (74.6)	131 (48.3)	94 (47.7)	35 (43.8)	.767
Prognosis					
GRACE score, median (IQR)	153.5 (131.0–179.8)	154.0 (130.0–179.0)	153.0 (133.0–180.0)	153.5 (127.5–180.8)	.928

Bold values are statistically significant (*P* < 0.05).

ACEI = angiotensin-converting enzyme inhibitor, ARB = angiotensin II receptor blocker, CAD = coronary artery disease, eGFR = estimated glomerular filtration rate, GRACE = Global Registry of Acute Coronary Events, IQR = interquartile range, LVEF = left ventricular ejection fraction, SD = standard deviation, STEMI = ST-segment elevation myocardial infarction.

### 3.2. Predictive value of *ACE* I/D for 12-month all-cause mortality in AMI patients

All-cause mortality at 12 months occurred in 58 cases (10.6%). Baseline characteristics differed between groups according to survival (Table 3). The group of patients who died were older (67.5 ± 10.5 vs 63.5 ± 11.7; *P* = .013) and also had a greater prevalence of diabetes mellitus (*P* = .010) and occurrence of Killip class ≥ II (*P* < .0001), as well as higher troponin I concentration at admission (*P* = .031) and higher GRACE scores (*P* < .0001). On the other hand, the deceased group had a lower estimated glomerular filtration rate (*P* < .0001), lower LVEF (*P* = .002), and less frequent application of ACEI/ARB therapy (*P* = .014) than the survivor group. There were no differences in the percentages of II, ID, and DD genotypes of the *ACE* I/D variant between the deceased and survivor groups.

**Table 3:** Comparative characteristics of study population according to survival status.

Variables	Death (n = 58)	Survivors (n = 490)	*P*
Clinical and laboratory parameters			
Age (yr), mean ± SD	67.5 ± 10.5	63.5 ± 11.7	**.013**
Male, n (%)	36 (62.1)	356 (72.7)	.091
Hypertension, n (%)	48 (82.8)	400 (81.6)	.834
Diabetes mellitus, n (%)	22 (37.9)	111 (22.7)	**.010**
Dyslipidemia, n (%)	52 (89.7)	437 (79.7)	.913
Obesity, n (%)	10 (17.2)	102 (20.8)	.523
Smoking, n (%)	22 (37.9)	211 (43.1)	.455
Family history of premature CAD, n (%)	4 (6.9)	33 (6.7)	1.000
STEMI, n (%)	38 (65.5)	313 (63.9)	.806
Killip Class ≥ II, n (%)	29 (50.0)	100 (20.4)	**<.0001**
Troponin I on admission (pg/mL), median (IQR)	37.5 (3.3–50.0)	14.2 (2.9–50.0)	**.031**
eGFR (mL/min/1.73 m^2^), median (IQR)	62.1 (40.5–86.7)	84.5 (67.4–94.8)	**<.0001**
LVEF (%), median (IQR)	40.5 (31.5–50.0)	47.0 (40.0–53.0)	**.002**
Coronary lesions			
Single vessel disease, n (%)	12 (20.7)	121 (24.7)	.672
Double vessel disease, n (%)	23 (39.7)	168 (34.3)	
Triple vessel disease, n (%)	23 (39.7)	201 (41.0)	
Left main disease, n (%)	7 (12.1)	47 (9.6)	.549
Gensini score, median (IQR)	45.5 (18.8–68.0)	34.0 (17.0–57.0)	.150
*ACE* I/D genotype			
II, n (%)	24 (41.4)	247 (50.4)	.403
ID, n (%)	25 (43.1)	172 (35.1)	
DD, n (%)	9 (15.5)	71 (14.5)	
Medications at discharge			
Aspirin, n (%)	57 (98.3)	490 (100.0)	.106
P2Y12 inhibitor, n (%)	58 (100.0)	490 (100.0)	-
Statins, n (%)	57 (98.3)	483 (98.6)	.594
ACEI/ARB, n (%)	47 (81.0)	447 (91.2)	**.014**
Beta-blocker, n (%)	22 (37.9)	238 (48.6)	.125
Prognosis			
GRACE score, median (IQR)	177.0 (156.0–206.8)	150.0 (129.0–173.0)	**<.0001**

Bold values are statistically significant (*P* < 0.05).

ACEI = angiotensin-converting enzyme inhibitor, ARB = angiotensin II receptor blocker, CAD = coronary artery disease, eGFR = estimated glomerular filtration rate, GRACE = Global Registry of Acute Coronary Events, I/D = insertion/deletion, IQR = interquartile range, LVEF = left ventricular ejection fraction, SD = standard deviation, STEMI = ST-segment elevation myocardial infarction.

The association between *ACE* I/D genotype and 12-month all-cause mortality in patients with AMI was analyzed using Cox regression with different genetic models (Table 4). The results did not show an effect of the *ACE* I/D variant on all-cause mortality in the study population.

**Table 4:** Cox regression analysis for 12-mo all-cause mortality in different genetic models.

Genetic models	Genotypes	HR	95% CI	*P*
Dominant model				
	II	Ref		
	ID/DD	1.41	0.84–2.38	.196
Recessive model				
	II/ID	Ref		
	DD	1.08	0.53–2.20	.827
Homozygous model				
	II	Ref		
	DD	1.29	0.60–2.78	.515
Heterozygous model				
	II	Ref		
	ID	1.46	0.84–2.56	.184

CI = confidence interval, HR = hazard ratio.

**Figure 1.**
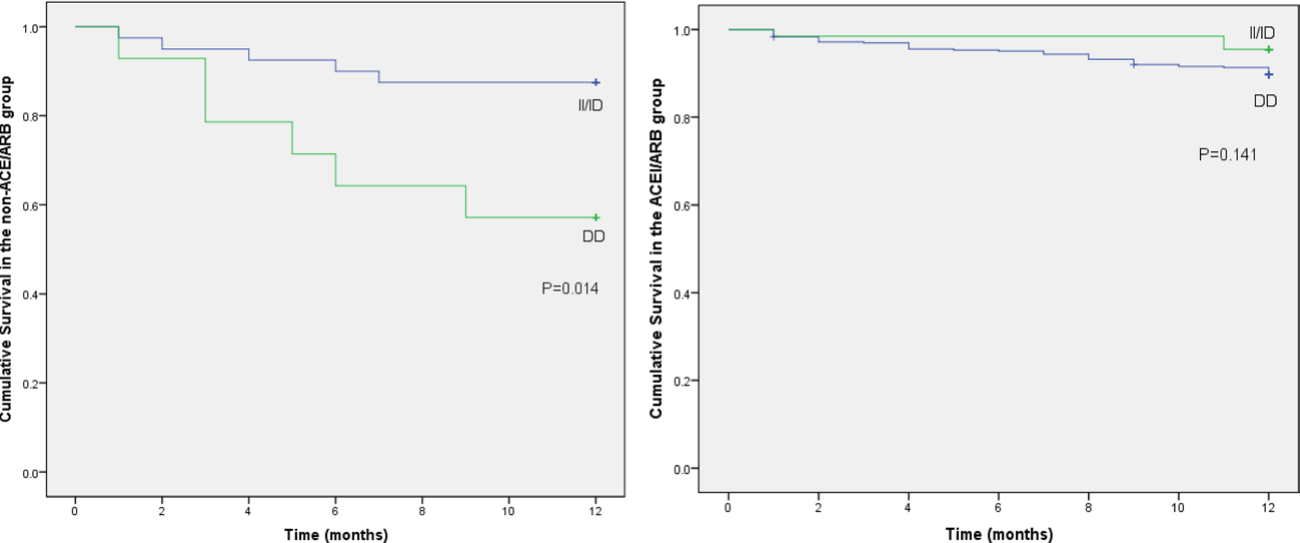
Kaplan–Meier curves of cumulative survival in AMI patients with or without ACEI/ARB therapy. ACEI = angiotensin-converting enzyme inhibitor, AMI = acute myocardial infarction, ARB = angiotensin II receptor blocker.

A subgroup analysis by the usage status of ACEI/ARB showed that in the non-ACEI/ARB group, patients with the DD genotype had a lower cumulative survival probability than patients with II or ID genotypes (HR = 3.97; 95% confidence interval [CI]: 1.21–13.04; *P* = .023). In the group of patients who did not use ACEI/ARB during the 12 months of follow-up, 6 of the 14 DD-carrying patients died from all causes (42.9%) compared with 5 of the 40 II/ID-carrying patients (12.5%) (log-rank test *P* = .014). On the other hand, there was no association between *ACE* I/D genetic polymorphism and all-cause mortality in the ACEI/ARB group (log-rank test *P* = .141) (Fig. 1).

**Figure 2.**
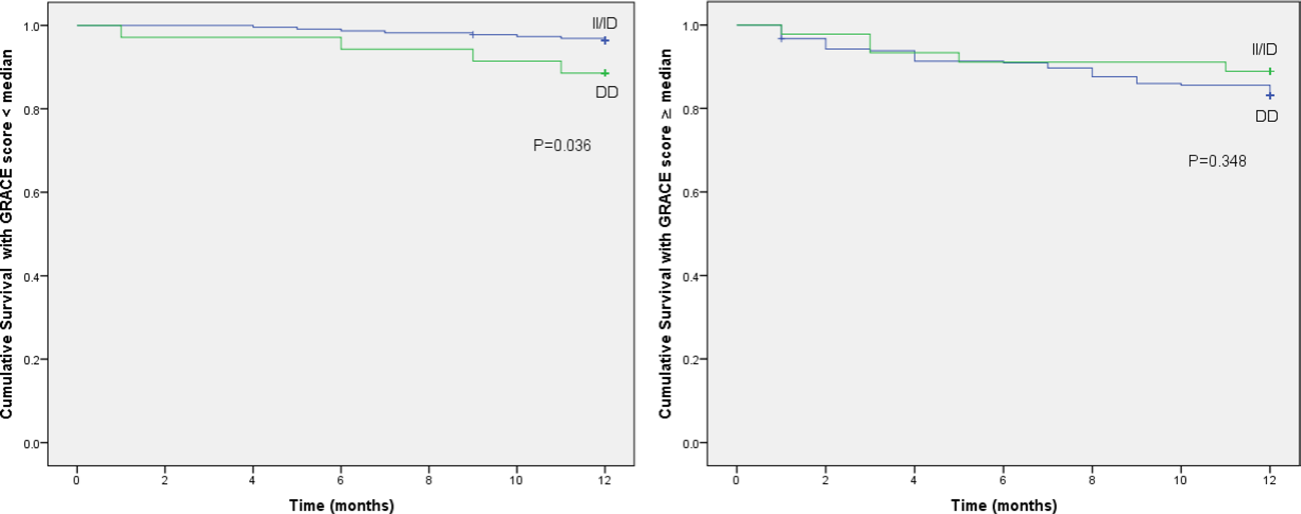
Kaplan–Meier curves of cumulative survival in AMI patients with GRACE score less than or equal to and greater than median. AMI = acute myocardial infarction, GRACE = Global Registry of Acute Coronary Events.

In regard to subgroup analysis by GRACE score on admission, we found that in the group of patients with a GRACE score below the median (153.5 points), patients carrying the DD genotype had a lower probability of cumulative survival than those carrying the II or ID genotype (HR = 3.35; 95% CI: 1.01–11.11; *P* = .049). Specifically, in the group with a GRACE score < 153.5 points, 4 of the 35 patients with the DD genotype died from any cause (11.4%) compared with 8 of the 225 patients carrying the II or ID genotype (5.6%) (log-rank test *P* = .036). In contrast, in the group with a GRACE score equal to or greater than the median, there was no statistically significant difference in 12-month all-cause mortality between DD-carrying patients and II/ID-carrying patients (log-rank test *P* = .348) (Fig. 2).

## 4. Discussion

The distribution of *ACE* I/D genotypes in this study did not comply with the Hardy-Weinberg equilibrium. This could be explained by the fact that our study included only pathological population. The deviation from Hardy-Weinberg equilibrium could also result from selection bias or genotyping error. Nevertheless, selection bias was tightly controlled by selecting AMI subjects accurately who satisfied the sampling criteria through careful history taking, thorough clinical examination, and performing all necessary tests to diagnose AMI definitely. In addition, the genetic testing in our study was performed according to precise and rigorous procedures.

There was no control group in our study because it might not be needed to answer the research question of whether *ACE* I/D genetic polymorphism was associated with 12-month all-cause mortality in AMI patients. Similarly, several previous studies also did not recruit a control group.^[8,9,19]^ Patients were followed up for 12 months from the date of admission and were found to have an all-cause mortality rate of 10.6%. Patients after AMI are at risk of various major adverse cardiovascular events, including the risk of death. The post-infarction 1-year mortality rate typically ranges from 10% to 12%.^[2,26]^ However, the risk of mortality in patients with AMI could be reduced by taking advantage of recent advances in coronary revascularization, medical therapy, and lifestyle modifications.^[2]^

Several studies in Kinh Vietnamese population have shown the important role of genetic factor in cardiometabolic diseases and their outcomes.^[27–29]^ Our study did not detect an association between the *ACE* I/D variant and all-cause mortality in the studied population using different genetic models. The results are consistent with other studies.^[16–19,30]^ Moorthy N et al^[19]^ conducted a study of 934 Indian patients with STEMI who were treated with fibrinolysis and found no association between the *ACE* I/D polymorphism and in-hospital mortality. Similarly, Samani NJ et al^[16]^ found with a median follow-up of 15 months (3–22 months) of 684 patients with AMI, overall mortality was not different between patients with the DD and ID/II genotypes. Regarding long-term prognosis and the *ACE* I/D variant, Keavney B et al^[17]^ concluded from a 5-year follow-up of 4269 AMI patients in the ISIS-3 study that there was no difference in the effect of the II, ID and DD genotypes.

In contrast, the I/D polymorphism of the *ACE* gene has been reported in several studies to influence mortality in AMI patients.^[8–11,31]^ Evans AE et al^[31]^ reported on a study of 213 deaths with definite or possible AMI as part of the Belfast MONICA Project in 1994 that the D allele and the DD genotype are associated with mortality. A study by Chen YH et al^[11]^ showed that in Taiwanese patients with acute coronary syndrome, the DD genotype increases the risk of in-hospital sudden cardiac death. The DD genotype was also found in a study in Japan using multivariate analysis adjusting for confounders and a mean follow-up of 9.4 months to be related to mortality in AMI patients.^[8]^ In addition, DD/ID genotypes were shown with a mean follow-up of 2.6 years to be an independent predictor of death for AMI patients in New Zealand.^[9]^

The reason for the differences in the prognostic role of the *ACE* I/D polymorphism in various studies could be disparities in genetic characteristics between different races, sample size, length of follow-up, and the usage of standard ACEI/ARB therapy. The original observation by Cambien et al linked the DD genotype to AMI risk but did not address AMI prognosis.^[13]^ The issue of association between *ACE* I/D genetic polymorphism and prognosis came later in subsequent studies and remains especially controversial. Accordingly, the AMI population selected in the present study might already be enriched in AMI susceptibility genes that made the effect of the DD genotype on mortality less or not apparent. ACEI/ARB drugs have been shown in previous studies to improve mortality in patients after AMI.^[32–34]^ The effect of ACEI/ARB could obscure the predictive value of the *ACE* I/D variant. Therefore, we performed a subgroup analysis in AMI patients with and without ACEI/ARB therapy. In this study, in the non-ACEI/ARB group, patients carrying the DD genotype had a 3.879-fold increased risk of 1-year mortality compared with those carrying the II/ID genotypes. However, in the ACEI/ARB group, the *ACE* I/D variant was not associated with all-cause mortality. The proportion using ACEI/ARB drugs in our study was 90.1%. This rate was much higher than in studies conducted by Palmer BR et al,^[9]^ Goldenberg I et al^[35]^ and Hara M et al.^[36]^ However, these studies did not analyze the association between *ACE* I/D polymorphism and mortality in groups with and without ACEI/ARB therapy.

In our study, among AMI patients with a GRACE score on admission of less than the median (153.5 points) and carrying the DD genotype, the risk of 12-month mortality increased by 3.346 times compared with those with the II/ID genotypes. The association between the *ACE* I/D variant and mortality was not statistically significant in the group with GRACE scores equal to or greater than the median. This was a novel finding because other studies have not performed the subgroup analysis by GRACE score. This result suggests that in AMI patients whose mortality risk is not high according to the GRACE score, the predictive value of the *ACE* I/D variant is more obvious. On the other hand, in high-risk AMI patients with above-median GRACE scores, the non-statistically significant effect of the *ACE* I/D variant on mortality might be due to the prevalence of many common risk factors, various comorbidities, complex clinical presentations, and aggressive treatment strategies.

There were several limitations to this study. First, serum ACE concentrations were not measured and evaluated for the effect on mortality. Second, this was an observational study, so it did not interfere with patient adherence to therapy, which might affect outcomes during follow-up. Third, this study was conducted in a single center, thus it might not be representative of the characteristics of AMI and the genotypes of the *ACE* I/D polymorphism in the entire Vietnamese population. Finally, although our study had enough statistical power in detecting the differences in mortality between DD and II/ID genotypes in subgroups including patients who did not use ACEI/ARB (statistical power = 96.1%) and patients with a GRACE score below the median (statistical power = 100.0%) but the sample size might not be enough to detect lower level of differences, especially in the general AMI population. Therefore, further studies with a larger sample size of AMI patients are needed to confirm the association between *ACE* I/D genetic polymorphism and all-cause mortality.

In conclusion, the *ACE* I/D genetic polymorphism was not found to be associated with 12-month all-cause mortality in patients with AMI. However, in groups without ACEI/ARB therapy or with GRACE scores below 153.5 points, patients with the DD genotype had a higher risk of all-cause mortality than those carrying the II/ID genotypes. This is the first study in Vietnam investigating the prognostic role of the *ACE* I/D variant in AMI patients and it may offer valuable information for secondary prevention strategies in subjects with AMI.

## Author contributions

**Conceptualization:** Duy Cong Tran, Minh Duc Do, Binh Quang Truong.

**Data curation:** Duy Cong Tran, Truc Thanh Thai.

**Formal analysis:** Duy Cong Tran, Minh Duc Do, Linh Hoang Gia Le, Truc Thanh Thai, Sy Van Hoang, Binh Quang Truong.

**Funding acquisition:** Duy Cong Tran.

**Investigation:** Duy Cong Tran, Minh Duc Do, Linh Hoang Gia Le.

**Methodology:** Duy Cong Tran.

**Project administration:** Duy Cong Tran, Binh Quang Truong.

**Resources:** Duy Cong Tran.

**Visualization:** Duy Cong Tran.

**Writing – original draft:** Duy Cong Tran, Minh Duc Do, Linh Hoang Gia Le, Truc Thanh Thai, Sy Van Hoang, Binh Quang Truong.

**Writing – review & editing:** Duy Cong Tran, Minh Duc Do, Sy Van Hoang, Binh Quang Truong.
